# Medical students’ health behaviour and self-reported mental health status by their country of origin: a cross-sectional study

**DOI:** 10.1186/s12888-016-0884-8

**Published:** 2016-05-28

**Authors:** András Terebessy, Edit Czeglédi, Bettina Claudia Balla, Ferenc Horváth, Péter Balázs

**Affiliations:** Department of Public Health, Faculty of Medicine, Semmelweis University, Nagyvárad tér 4, H-1089 Budapest, Hungary; Department of Behavioural Sciences, Faculty of Medicine, Semmelweis University, Nagyvárad tér 4, H-1089 Budapest, Hungary

**Keywords:** Mental health, Medical students, Alcohol consumption, Exercise, Country of origin

## Abstract

**Background:**

Numerous previous studies have investigated the lifestyle and self-perceived health status of medical students. This study examined whether students’ country of origin contributed to their mental health and health risk behaviour.

**Methods:**

We conducted our cross-sectional questionnaire survey over four consecutive years (2009–2012). The target population was fourth-year English- and Hungarian-language course medical students at Semmelweis University, Hungary. We gathered data on medical students’ health behaviour (tobacco smoking, alcohol consumption, dietary habits and exercise) and mental health status and used analysis of variance (ANOVA) to examine the association between country of origin and mental health.

**Results:**

The response rate was 76.1 % for the Hungarian and 63.4 % for the English course students. The mean age of our sample was 24.1 years (SD = 2.42). Only 15.3 % of students reported following dietary recommendations, but 75.0 % reported engaging in vigorous and regular physical exercise. The prevalence of tobacco smoking was 18.6 % and 13.8 % overconsumed alcoholic beverages. Hungarian and Iranian students reported lower mental well-being than Mediterranean, Israeli and Scandinavian students (F_(4)_ = 18.943, *p* < 0.001, η^2^ = 0.103). Results of the multiway ANOVA indicated that both country of origin and exercise showed a significant relationship with mental health: vigorous exercise was associated with better mental health status (F_(1)_ = 5.505, *p* = 0.019).

**Conclusions:**

Medical students’ mental health and health behaviour are associated with multiple factors. One of these is country of origin but exercise may also influence mental health. Health promotion programmes organised for medical students should take their country of origin into consideration and should include physical activity.

## Background

Physicians care not only for their patients’ health but also support their patients in coping with lifestyle problems. Health-care providers can act as role models by setting an example of healthy behaviour and a healthy lifestyle for their patients [1]. Some studies have demonstrated a link between medical doctors’ physical activity levels and their motivation to encourage patients to undertake intensive physical exercise. Doctors with alcoholic problems underestimate the relevance and necessary frequency of alcohol counselling [2]. Earlier studies found an association between medical students’ health behaviour and their plans for counselling activities [3, 4]. Other findings indicate that patterns of health-related behaviour in medical students are created even before they enter higher education [3–6].

Pre-existing unhealthy behavioural patterns may continue to develop during students’ medical training. Students in later academic years show a higher prevalence of drinking, smoking and substance abuse [7] and their consumption of fruit and vegetables declines with every completed semester [8]. The academic environment may affect the mental health of college students; for example, academic overload may lead to stress, anxiety and depression [9]. Contact with patients in poor health can also be a unique stressor in medical school. The incidence of mental health problems among medical students may be as high as 30 %, but most students do not seek help [10]. This may explain why students tend to use alternative methods—like alcohol consumption—to cope with stress.

Cultural background may also influence health risk behaviours and self-rated health status [11]. Immigration flows are on the rise in most OECD countries and the number of migrant doctors working in these countries elevated with 60 % since 2004. There are some important variations among OECD countries in the proportion of health personnel born abroad. More than 30 % of practising medical doctors in the United Kingdom, Switzerland, Sweden and Canada are foreign-born but Norway, Belgium and the United States also exceed 20 %. The phenomenon can also be observed in Central-Eastern European countries as well: the share of foreign-born physicians exceeds 10 % in Slovenia and Hungary and is around 3 % in Poland and Slovakia [12]. Some medical schools in the world are training doctors for their home country as well as for the international labour market [13]. Learning experiences of international medical graduates must be individualized because residents come from very different cultures with different values [14]. Studying abroad in a foreign language environment is a challenge that cannot be underestimated [15]. Norwegian medical students studying abroad smoke more frequently and consume alcohol more excessively than their domestic peers [16]. Comparisons of culturally different populations may help to explain factors related to health behaviours in students. Numerous studies have attempted to assess medical students’ lifestyle and health status, but only a few studies have compared samples from different cultures [9, 16–18].

The aim of our study was to explore the health behaviour (i.e., nutrition, exercise, tobacco smoking and alcohol consumption) and certain aspects of mental health (i.e., role limitations because of emotional problems, energy/fatigue, emotional well-being and social functioning) of medical students in Hungary. Our study also aimed to compare foreign and Hungarian students in this regard.

## Methods

### Participants

A repeated cross-sectional questionnaire survey was conducted at the Faculty of Medicine of Semmelweis University, Budapest, Hungary. Our target population was students in their fourth academic year between 2009 and 2012. We invited English training programme students to participate from the academic year 2009/2010; Hungarian students were also included from 2011/2012. In practical terms, the only difference between the Hungarian and the foreign students’ curricula was the training language. According to the academic administration system’s electronic register, the total target population was 1683 (878 Hungarian and 805 English course students). All students on the English course were invited to participate and half of the Hungarian study groups (20 groups, 351 students in total) were randomly sampled. We distributed the paper-based questionnaires at the end of the Public Health seminaries. We set up five subsamples by participants’ country of origin: 1) European Mediterranean countries (Spain, France, Italy, Greece and Cyprus); 2) Scandinavian countries (Norway and Sweden); 3) Israel; 4) the Islamic Republic of Iran and 5) Hungary.

### Measurement instrument

The first part of the questionnaire collected basic demographic data like sex, age and country of origin. Diet and exercise were measured by the Simple Lifestyle Indicator Questionnaire (adapted by the authors to produce a Hungarian version) [19]. Diet was measured using three questions about the consumption of vegetables, fruits and high-fibre cereals with six response options ranging from 0 (less than once a week) to 5 (two or more times per day). The total possible score for diet on this scale ranged from 0 to 15. Following the scoring method of the original scale, the score ranges 0–5, 6–10 and 11–15 were categorised as 1, 2 and 3 (‘unhealthy’, ‘average’ and ‘healthy’), respectively [19].

Exercise was measured using three questions about the frequency of light, moderate and vigorous exercise. Response options to these questions were: never, 1–2 times a week, 3–4 times a week and 5–7 times a week. Responses were categorised as 1, 2 and 3 (‘inactive’, ‘moderately active’ and ‘active’), representing 1) reported light exercise only, 2) some moderate activity (but no vigorous activity) and 3) any kind of vigorous activity, respectively [19].

Smoking was assessed using the six-item Fagerström Test for Nicotine Dependence. The overall potential score ranges from 0 to 10; with higher scores indicating more dependence. For more information about this test, please see the original reference [20].

Participants indicated how many standard drinks of beer, wine and spirits they consumed in an average week. A standard drink was defined as an alcoholic beverage containing approximately 10 g of pure alcohol. Overconsumption of alcohol was defined as seven or more standard drinks for women, and 14 or more for men per week, based on the recommendations of the Dietary Guidelines for Americans, 2010 [21].

The Short Form Health Survey (SF-36) was used to assess self-reported mental health because of its good psychometric properties. The SF-36 is a 36-item scale constructed to survey health status and quality of life. The SF-36 assesses eight health concepts: limitations in physical activities because of health problems; limitations in social activities because of physical or emotional problems; limitations in usual role activities because of physical health problems; bodily pain; general mental well-being; limitations in usual role activities because of emotional problems; vitality; and general health perceptions. Responses are made on Likert-type scales, some with 5 or 6 points and others with 2 or 3 points. For each domain, an aggregated score is produced ranging from 0 (lowest or worst possible level of functioning) to 100 (highest or best possible level of functioning). For more information about the SF-36, please see the original study [22]. In this study, we analysed only four subscales referring to mental health. The ‘Role limitation due to emotional problems’ subscale describes to what extent emotional problems interfere with accomplishments at work or other usual activities in terms of time, as well as performance. The ‘Vitality’ subscale measures subjective well-being ratings in terms of energy and fatigue. The ‘Social functioning’ subscale measures normal social functioning due specifically to health-related problems. The ‘Emotional well-being’ subscale provides information about the responders’ overall psychological wellbeing representing major mental health dimensions [23]. Internal consistency of the subscales proved to be acceptable. The Cronbach-alpha coefficients of the scales for the current study sample ranged from 0.75 to 0.83.

### Statistical analysis

We used IBM-SPSS 21.0 for Windows for all analyses (Armonk, NY: IBM Corporation). Individuals refusing to answer specific questions were excluded only from evaluation of the question concerned. Prevalence was measured as percentages; averages were means (with standard deviations). To assess the internal consistency of the scales, we calculated Cronbach’s alpha values with 95 % confidence intervals (CIs). Binary data (either as collected or collapsed) were analysed using the chi-square test and binary logistic regression analysis with odds ratios (ORs) with 95 % CIs at a significance level of *p* < 0.05. For the chi-square analyses, we used Cramer’s V to calculate the effect size; effect sizes were interpreted as follows: small effect (0.1), medium effect (0.3) and large effect (0.5) [24]. One-way analysis of variance (ANOVA) was performed to compare mental health subscales outcomes among country groups. We used the Games–Howell test for post-hoc analysis. When violations of normality assumptions were detected, Kruskal–Wallis tests were implemented. Principal component analysis was performed by aggregating the four SF-36 mental health subscales. To test the relationship between country of origin and mental well-being, we used a multiway ANOVA after adjusting for potential confounding variables (gender and age), and health-related behaviours and variables (diet, exercise, alcohol use, nicotine dependence).

## Results

### Analysis of demographic data

Overall, 777 individuals participated in the survey (267 domestic and 510 foreign students), representing a response rate of 76.1 and 63.4 % respectively. We classified 629 students (267 men, 341 women, 21 unknown) into five subsamples by their country of origin (analysis of a greater range of countries was not possible because of the low number of respondents from particular countries). Table 1 shows the demographic data and characteristics of health (and health risk) behaviour of these subsamples. Israeli and Iranian students were older than Mediterranean, Scandinavian and Hungarian students. There were significant differences in gender distribution. The Hungarian subsample contained the highest proportion of females and the Israeli subsample the lowest. We categorised 15.3 % of participants as diet category 3 (‘healthy diet’) with no significant differences among subsamples. Seventy-five per cent of participants reported regularly performed vigorous exercise and Hungarians and Scandinavians reported performing this type of physical activity more than did Mediterranean students. The overall prevalence of tobacco smoking was 18.6 %. Prevalence was highest among Mediterranean students. The overall reported prevalence of alcohol overconsumption was 13.8 %. Scandinavian students’ weekly consumption was higher than that of any other subsample and Hungarians reported the lowest amount of alcohol consumed.

**Table 1:** Descriptive statistics of demographic data and health behaviour

Variables		Total	Mediterranean	Scandinavian	Israel	Iran	Hungary	Group differences
Gender N (%)	Males	267 (43.9)	37 (54.4)	54 (40.9)	65 (66.3)	23 (54.8)	88 (32.8)	χ^2^_(4)_ = 38.874*** (Cramer’s V = 0.253*)
	Females	341 (56.1)	31 (45.6)	78 (59.1)	33 (33.7)	19 (45.2)	180 (67.2)	
Age (unit: years) Mean (SD)		24.1 (2.42)	22.9^b,c,d^ (1.94)	24.8^a,c,e^ (2.21)	26.3^a,b,e^ (1.94)	26.1^a,e^ (3.06)	22.8^b,c,d^ (1.46)	H_(4)_ = 233.429*** (η^2^ = 0.356)
Diet N (%)	Unhealthy	200 (33.2)	24 (36.4)	39 (28.1)	35 (37.2)	11 (25.0)	91 (35.0)	χ^2^_(8)_ = 8.416 (Cramer’s V = 0.084)
	Average	311 (51.6)	33 (50.0)	71 (51.1)	45 (47.9)	28 (63.6)	134 (51.5)	
	Healthy	92 (15.3)	9 (13.6)	29 (20.9)	14 (14.9)	5 (11.4)	35 (13.5)	
Exercise N (%)	Inactive	74 (11.8)	16 (22.9)	13 (9.2)	10 (9.7)	9 (20.0)	26 (9.7)	χ^2^_(8)_ = 22.636** (Cramer’s V = 0.134**)
	Moderately active	83 (13.2)	15 (21.4)	13 (9.2)	16 (15.5)	5 (11.1)	34 (12.7)	
	Active	470 (75.0)	39 (55.7)	115 (81.6)	77 (74.8)	31 (68.9)	208 (77.6)	
Smoker N (%)		115 (18.6)	21 (30.4)	17 (12.4)	22 (21.4)	7 (16.7)	48 (17.9)	χ^2^_(4)_ = 10.567* (Cramer’s V = 0.131*)
Nicotine dependence of smokers Mean (SD)		1.7 (1.89)	2.4 (1.86)	1.4 (2.26)	1.8 (1.78)	3.3 (3.45)	1.2 (1.24)	H_(4)_ = 7.601 (η^2^ = 0.102)
Alcohol consumption (unit: standard drinks) Mean (SD)		5.0 (6.67)	6.7^e^ (8.25)	8.1^c,d,e^ (8.41)	4.6^b^ (5.85)	3.9^b^ (4.20)	3.3^a,b^ (4.91)	H_(4)_ = 74.218*** (η^2^ = 0.090)
Overconsumption of alcohol N (%)		81 (13.8)	12 (18.5)	41 (32.0)	7 (7.4)	3 (8.6)	18 (6.8)	χ^2^_(4)_ = 51.889*** (Cramer’s V = 0.297***)

Note: *p < 0.05, **p < 0.01, ***p < 0.001. ^a^Mediterranean, ^b^Scandinavian, ^c^Israel, ^d^Iran, ^e^Hungary. Upper indexes in cells indicate significant mean difference from certain subsamples

### Predictors of health behaviour

A multivariate binary logistic regression model was used to test for predictors of good dietary practice, high levels of exercise, smoking and overconsumption of alcohol. The diet categories 2 and 3 (‘average’ and ‘healthy’) were collapsed to separate students who followed an unhealthy diet (*n* = 200) from those who followed guideline recommendations at least partially (*n* = 403). Exercise categories 1 and 2 (‘inactive’ and ‘moderately active’) were also collapsed to separate students engaging in insufficient levels of physical exercise (*n* = 157) from those engaging in high levels of exercise (*n* = 470). Mediterranean students were the general reference group during the analyses. Females reported following healthy diet more likely than males, but males reported engaging in vigorous exercise more likely than females. The odds for smoking were lower among Scandinavian students than Mediterranean students; however, Israeli and Iranian students were less likely to smoke than their Mediterranean peers at a tendency level. Being Scandinavian was a predictor for alcohol overconsumption while Hungarian students drank less likely than Mediterranean students. Age was also significantly associated with alcohol consumption: younger participants were more likely to drink excessively than older ones (Table 2).

**Table 2:** Predictors of good dietary practice, high level of exercise, smoking and overconsumption of alcohol

Predictors		Good dietary practice (*N* = 553)		High level of exercise (*N* = 570)		Smoking (*N* = 563)		Overconsumption of alcohol (*N* = 553)	
		OR (CI_95_)	*p*-value	OR (CI_95_)	*p*-value	OR (CI_95_)	*p*-value	OR (CI_95_)	*p*-value
Country group (ref: Mediterranean)	Scandinavian	1.38 (0.57–3.34)	0.477	**4.17 (2.05–8.46)**	**<0.001**	**0.33 (0.15–0.72)**	**0.005**	**3.11 (1.39–6.93)**	**0.006**
	Israel	1.28 (0.47–3.52)	0.633	**2.24 (1.06–4.73)**	**0.034**	0.49 (0.22–1.10)	0.082	0.71 (0.24–2.12)	0.538
	Iran	0.88 (0.25–3.14)	0.843	1.83 (0.75–4.47)	0.185	0.36 (0.12–1.08)	0.069	0.75 (0.18–3.04)	0.684
	Hungary	0.77 (0.34–1.75)	0.537	**3.29 (1.82–5.96)**	**<0.001**	0.61 (0.32–1.15)	0.124	**0.31 (0.14–0.71)**	**0.006**
Gender (ref: men)		**2.38 (1.40–4.04)**	**0.001**	**0.57 (0.37–0.87)**	**0.009**	**0.56 (0.35–0.88)**	**0.011**	1.19 (0.70–2.02)	0.526
Age		0.99 (0.88–1.13)	0.928	0.97 (0.88–1.08)	0.606	1.05 (0.94–1.17)	0.414	**0.82 (0.71–0.94)**	**0.005**
Nagelkerke R^2^ (%)		4.5		6.3		4.8		16.7	

Note: Bold characters indicate significant (*p* < 0.05) association

### Country of origin and mental health status

The bivariate analyses showed that scores on the SF-36 mental health subscales differed significantly across the subsamples. Pairwise comparisons for the subscale ‘Role limitations due to emotional problems’ indicated that Hungarian students had significantly worse results (*p* < 0.01) than any other subgroup, but no significant differences were detected among the rest of the sample. Results were similar on the ‘Vitality’ subscale (*p* < 0.05), although the scores of Mediterranean, Iranian and Hungarian students did not differ significantly. Pairwise comparisons of ‘Emotional well-being’ subscale scores showed that Hungarian students scored significantly lower than Scandinavian and Israeli students (*p* < 0.05), while the scores of Iranian students were significantly lower than those of Scandinavian students (*p* = 0.003), and were marginally significantly lower than Israeli students’ scores (*p* = 0.053). Subsample differences were very similar for ‘Social functioning’ subscale scores: Iranian and Hungarian students had significantly lower mean scores than Israeli and Scandinavian students (*p* < 0.001) (Table 3.).

**Table 3:** Comparing countries by aspects of mental health

Scales of SF-36		Mean (SD)	95 % CI of mean	Median	Comparing groups
Role limitations due to emotional problems	Total	70.6 (39.10)	67.5–73.7	100	
	Mediterranean^e^	76.3 (39.23)	66.9–85.8	100	H_(4)_ = 67.420* (η^2^ = 0.095)
	Scandinavian^e^	79.7 (35.58)	73.7–85.6	100	
	Israel^e^	85.8 (29.57)	80.0–91.6	100	
	Iran^e^	80.0 (33.63)	69.9–90.1	100	
	Hungary^a,b,c,d^	57.1 (40.74)	52.2–62.0	66.7	
Vitality	Total	58.4 (19.34)	56.9–59.9	60	
	Mediterranean	58.9 (17.53)	54.7–63.1	60	F_(4)_ = 8.974* (η^2^ = 0.055)
	Scandinavian^e^	63.7 (17.63)	60.8–66.7	65	
	Israel^e^	63.5 (17.36)	60.1–66.9	65	
	Iran	58.0 (17.26)	52.7–63.2	60	
	Hungary^b,c^	53.6 (20.53)	51.2–56.1	55	
Emotional well-being	Total	72.2 (17.86)	70.8–73.6	76	
	Mediterranean	72.9 (15.90)	69.1–76.8	76	F_(4)_ = 12.641* (η^2^ = 0.074)
	Scandinavian^d,e^	78.8 (15.29)	76.3–81.4	84	
	Israel^e^	76.4 (14.03)	73.6–79.1	80	
	Iran^b^	69.3 (14.35)	64.9–73.6	70	
	Hungary^b,c^	67.3 (19.86)	65.0–69.7	72	
Social functioning	Total	77.3 (23.02)	75.5–79.1	87.5	
	Mediterranean	75.4 (24.82)	69.4–81.3	87.5	H_(4)_ = 45.698* (η^2^ = 0.065)
	Scandinavian^d,e^	83.7 (21.18)	80.2–87.2	87.5	
	Israel^d,e^	85.5 (17.21)	82.2–88.9	87.5	
	Iran^b,c^	71.3 (20.63)	65.0–77.6	75	
	Hungary^b,c^	72.2 (24.15)	69.3–75.1	75	

Note: *p < 0.001. ^a^Mediterranean, ^b^Scandinavian, ^c^Israel, ^d^Iran, ^e^Hungary. Upper indexes in cells indicate significant mean difference between certain subsamples

The principal component analysis of the SF-36 mental health subscales produced one principal component. Component loadings of the subscales ranged from 0.76 to 0.89 and 65.9 % of the total variance was explained. Results of the one-way ANOVA indicated that country of origin was significantly related to mental health (F(4) = 18.943, *p* < 0.001, η^2^ = 0.103). Post-hoc tests (the Games–Howell test) indicated that the mental well-being of Hungarian students was significantly lower than that of students from other countries (except Iran).

**Fig. 1:**
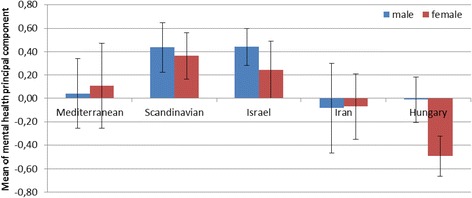
Comparing countries by mental health principal component by gender. Note: 95 % CIs of means are shown in the figure

Results of multiway ANOVA indicated a significant main effect of country of origin on mental health (F(4) = 7.780, *p* < 0.001) after adjusting for health behaviours, health risk behaviours and potential confounding variables. Vigorous exercise was associated with better mental health (F(1) = 5.505, *p* = 0.019) while diet and gender showed marginally significant main effects (F(1) = 3.080, *p* = 0.080, and F(1) = 3.081, *p* = 0.080, respectively). Alcohol consumption (F(1) = 0.095), nicotine dependence (F(1) = 0.137) and age (F(1) = 0.050) showed no significant association with mental health. The country of origin and gender interaction proved to be marginally significant (F(4) = 2.134, *p* = 0.075). Figure 1 demonstrates that the mental well-being of female and male Mediterranean, Scandinavian, Israeli and Iranian students is quite similar. In case of Hungarian students, female mental well-being scores are lower than those of males. The model explained 14.4 % of the total variance.

## Discussion

Previous studies have indicated that medical training is more stressful than other types of education [25], although some recent research suggests that attending medical school is not associated with increased stress [26]. However, stress at university may have a debilitating effect on studying [27] and can even result in burnout [28], suicidal thoughts and suicide among medical students [29]. Understanding medical students’ lifestyle habits and mental health may be a first step to the stress prevention and the improvement of quality of life.

We found that only 15.3 % of our sample reported ‘healthy diet’ that meets dietary recommendations at least partially [21, 30] and the majority (51.6 %) consumed fruit and vegetables only 3–4 times a week. These results are not surprising. Most of our sample was from European countries with insufficient vegetable consumption [31]. Women were more likely to report ‘healthy diet’ than men, and this finding agrees with previous scientific results [32–34]. Country of origin did not seem to be related to healthy dietary practices and we concluded that students from Mediterranean countries do not necessarily follow the Mediterranean diet. Previous studies have highlighted recent dietary changes in Mediterranean countries; namely, an increase in the intake of processed foods and saturated fat and a decrease in the intake of plant foods and monounsaturated fatty acids [35, 36]. In our sample, 20.9 % of Scandinavian students reported good dietary practice. Based on personal communications from the students, we hypothesise that food availability could explain this pattern. In Hungary (where students live during their academic years), fruit and vegetables are much cheaper than in their home countries. Our participants were medical students and part of their training focused on the advantages of a healthy diet. As obesity is a modern worldwide epidemic, the dietary behaviour patterns found here need to be changed (and not only for the personal health of our medical students). Physicians who are healthy are more credible to patients who consult them about prevention of illness [1] and their dietary habits may help to bring about desired changes in their patients.

The overall prevalence of smoking was 18.6 % in our sample and was highest in the Mediterranean subsample (30.4 %). All subsamples showed lower prevalence than the general age-matched population of their home country [37–39] (the Mediterranean subsample showed an equal prevalence), which is promising. We assume this means that medical students understand the public health importance of not smoking, which will increase their credibility when advising smoking cessation for their future patients.

Seventy-five per cent of our sample engaged in regular vigorous exercise (males more often than females). These findings support previous findings [33, 34]. Our results suggest that Mediterranean and Iranian students are the least physically active. Iranian researchers have found the same low levels of exercise in their homeland population [40, 41]. It is recommended that everyone should perform daily exercise; such activity helps future medical doctors to promote physical activity to their patients [3].

The overall prevalence of alcohol overconsumption was 13.8 %. There was a strong link between being Scandinavian and alcohol overconsumption for both genders. According to the World Economic Forum’s Global Gender Gap Report, 2013, Norway and Sweden were ranked third and fourth in terms of general gender equality (in comparison, Hungary ranked 87th on the same list). Unfortunately, there may be equality in bad habits too: women’s alcohol consumption patterns differ only a little from men’s in these countries. In addition, lifestyle may change when leaving the domestic cultural environment. A relevant former study indicated that Norwegian students behave differently in the domestic higher education system than abroad: in other countries, they seem to adopt the lifestyle of the host environment—particularly regarding alcohol-related behaviour [16]. Hungarian alcohol legislation is very permissive: people aged over 18 years may buy all kinds of alcoholic beverages without any restrictions; therefore, these products are available daily from the smallest grocery shops to the largest supermarkets. Additionally, alcoholic drinks are much cheaper in Hungary than in the Scandinavian countries. Informal communication with Scandinavian students (mostly Norwegians) revealed that, at the beginning of their studies, they arranged parties for social integration that involved frequent alcohol consumption.

Concerning mental health, the mean score of the ‘Vitality’ subscale of the four SF-36 subscales was the lowest in all subsamples. The lower scores on the ‘Vitality’ subscale may be a sign of fatigue in the fourth-year students’ population [28]. Normative data for the SF-36 are available for many countries so we could compare some of our results with previous findings (unfortunately, we could not find any normative data for Israel). For the Mediterranean subsample, we used the Greek data for comparison because this proportion was the highest. Scandinavian [42] and Iranian students’ [43] scores were about the same as those of their peers in their home country; Mediterranean students’ scores were a little lower [44], and Hungarian students’ scores much lower than their age-matched peers [45]. We can conclude that the self-reported mental health of our subsamples is better (or at least not worse) than reference values, excepting the Hungarian subsample. We should note that the last available normative data for the Hungarian population is from 1999 and there have been negative economic changes in Hungary since then. Therefore, the mental health of medical students (and the general population) in Hungary may have declined since 1999. Analysis of this phenomenon is beyond the scope of this paper.

Probably our most important finding is that the country of origin may affect mental health. Hungarian students rated their mental health significantly lower than the foreign student groups (with the best outcomes for Scandinavian and Israeli students). Country of origin was significantly associated with mental health even after adjusting for potential confounding variables (gender, age) and health (risk) behaviours. Although the association is significant it should be noted that the effect of the country of origin might be modest as only 5.5–9.5 % of the variance was explained by our models.

Our analysis of the potential predictors of mental health showed that regular, vigorous exercise predicted better mental health status. Regular physical activity has many health-related benefits, as it reduces the risk of cardiovascular diseases, certain cancers and diabetes and increases general well-being, generates positive mood and reduces symptoms of depression and anxiety [46]. The positive association between exercise and mental health is well known among young health-care professionals [47]. Motivating medical students to adopt an active lifestyle not only helps prevent ill-health, but also supports them in maintaining or improving their physical and mental health status.

As mental health may influence career options [48], it is crucial to improve medical students’ mental well-being so that they can begin their professional life without avoidable setbacks. There are several examples, even in Hungary, that demonstrate that self-development groups may facilitate coping with stress during academic training [49, 50].

There were some limitations of our study. Cross-sectional designs do not allow one to draw conclusions about cause–effect relationships. Voluntary participation might also result in bias. The size of some subsamples was small and reduced the power of certain statistical tests. Regarding the measures, we did not include a question about other forms of tobacco consumption (e.g., moist powder tobacco products, snus or water-pipe use). Additionally, the Simple Lifestyle Indicator Questionnaire contains only three questions about diet, which may not be enough to describe individuals’ dietary patterns. However we agree with the creators of this scale, who claimed that ‘people who have good dietary practices related to salads, fruit, and fibre also have good dietary practices around eating fish, not eating junk food, and choosing food low in saturated fat’ [19]. An additional limitation was that the purchase power of the students was not taken into consideration. Finally, there are four medical schools in our country, but we collected data only from a single school (although this was the largest); thus, we have to be cautious about generalizing from our results.

Despite limitations, we believe the study has some strength. We still do not have sufficient knowledge about the lifestyle and stamina of future medical doctors and the factors that may have a significant impact on these. To our knowledge, only a few prior studies have compared medical students by their country of origin. Our results indicated that country of origin could be a contributing factor to medical students’ mental health and health (risk) behaviours. These findings may add a small, yet important piece to the puzzle of understanding the factors underlying medical students’ well-being.

## Conclusion

Medical students’ health risk behaviour and mental health are associated with multiple factors. One of these may be students’ country of origin. Higher education programmes admit more and more students from very different parts of the world (shorter or longer visiting scholarships or even complete academic years spent abroad), creating a multicultural student community in many countries. Health promotion programmes for university students cannot ignore their country of origin and cultural background. Bad habits acquired earlier stay longer with us than good ones, and a permissive environment may reinforce this effect. Expanding the options for mental health training courses and continuous motivation to encourage medical students to adopt a physically active lifestyle may reduce the potentially adverse effects of their academic training.

## Abbreviations

ANOVA, analysis of variance; CI, confidence interval; OECD, Organisation for Economic Co-operation and Development; OR, odds ratio; SD, standard deviation; SF-36, Short Form Health Survey
